# Minimal mask immobilization with optical surface guidance for head and neck radiotherapy

**DOI:** 10.1002/acm2.12211

**Published:** 2017-11-09

**Authors:** Bo Zhao, Genevieve Maquilan, Steve Jiang, David L. Schwartz

**Affiliations:** ^1^ Department of Radiation Oncology The University of Texas Southwestern Medical Center Dallas TX USA; ^2^ Department of Radiation Oncology University of Tennessee Health Science Center‐West Cancer Center Memphis TN USA

**Keywords:** head and neck, image guidance, immobilization, interfraction motion, intrafraction motion, SGRT

## Abstract

**Purpose:**

Full face and neck thermoplastic masks provide standard‐of‐care immobilization for patients receiving H&N IMRT. However, these masks are uncomfortable and increase skin dose. The purpose of this pilot trial was to investigate the feasibility and setup accuracy of minimal face and neck mask immobilization with optical surface guidance.

**Methods:**

Twenty patients enrolled onto this IRB‐approved protocol. Patients were immobilized with masks securing only forehead and chin. Shoulder movement was restricted by either moldable cushion or hand held strap retractors. Positional information, including isocenter location and CT skin contours, were imported to a commercial surface image guidance system. Patients typically received standard‐of‐care IMRT to 60–70 Gy in 30–33 fractions. Patients were first set up to surface markings with optical image guidance referenced to regions of interest (ROIs) on simulation CT images. Positioning was confirmed by in‐room CBCT. Following six‐dimensional robotic couch correction, a new optical real‐time surface image was acquired to track intrafraction motion and to serve as a reference surface for setup at the next treatment fraction. Therapists manually recorded total treatment time as well as couch shifts based on kV imaging. Intrafractional ROI motion tracking was automatically recorded by the optical image guidance system. Patient comfort was assessed by self‐administered surveys.

**Results:**

Setup error was measured as six‐dimensional shifts (vertical/longitudinal/lateral/rotation/pitch/roll). Mean error values were −0.51 ± 2.42 mm, −0.49 ± 3.30 mm, 0.23 ± 2.58 mm, −0.15 ± 1.01^o^, −0.02 ± 1.19^o^, and 0.06 ± 1.08^o^, respectively. Average treatment time was 21.6 ± 8.4 mins). Subjective comfort during surface‐guided treatment was confirmed on patient surveys.

**Conclusion:**

These pilot results confirm feasibility of minimal mask immobilization combined with commercially available optical image guidance. Patient acceptance of minimal mask immobilization has been encouraging. Follow‐up validation, with direct comparison to standard mask immobilization, appears warranted.

## 1 INTRODUCTION

Patient immobilization is critical for safe, reproducible delivery of H&N radiotherapy. Thermoplastic masks routinely provide this immobilization. Many patients find masks constrictive and stressful. The density of thermoplastic is water equivalent and can create a skin bolus effect which intensifies skin reactions to treatment.^1^ Minimal open face masks may make treatment less uncomfortable and toxic for patients.

Three‐dimensional optical surface imaging can effectively monitor setup for surface‐guided radiation therapy (SGRT).^2^ SGRT is noninvasive and does not expose patients to ionizing radiation. Real‐time surface capture can be registered with a baseline reference surface, such as skin contours rendered from a CT or optical images taken at the time of treatment simulation. Displacement errors can be displayed in real time as six‐dimensional deltas to guide therapists during daily setup. Unlike online kV/MV imaging, SGRT provides continuous motion tracking during treatment. This has been leveraged to confirm breast setup accuracy during breath hold^3^, ^4^ and to track stereotactic treatment to cranial^5^, ^6^ and thoracic^7^ sites.

Several small series have quantified setup reproducibility of open face mask prototypes; however, these prototypes typically employed small openings limited to central face.^8^, ^9^ In contrast, we wished to more significantly reduce mask coverage to selectively immobilize only fulcrums of movement at the chin and forehead, and to validate optical surface guidance of these minimal mask setups via in‐room CBCT reference imaging.

## 2 METHODS

### 2.A SGRT platform and procedures

We used a commercial SGRT platform (AlignRT, Vision RT Ltd., London, UK) in all cases. The primary components of the system are three ceiling‐mounted optical camera units capable of capturing three‐dimensional real‐time surface data from the patient on the treatment couch.^2^ The cameras capture surface patterns projected onto patients, permitting *in silico* three‐dimensional surface renderings. We used an elevated grid phantom provided by the manufacturer to calibrate isocenter localization. Daily grid QA was performed with a threshold of 1 mm. LINAC imaging center coincidence was cross‐checked via an isocube phantom monthly.^10^ We set 1 mm/0.5° as a threshold to apply isocenter calibration correction to AlignRT.

### 2.B Study cohort

This study was approved by our institutional IRB with a target enrollment of 20 patients. Patients receiving curative head and neck radiotherapy requiring an extended thermoplastic mask to cover shoulders were eligible for enrollment. Patients in the study cohort are listed in Table 1. All patients received standard‐of‐care IMRT to 60–70 Gy in 30–33 daily fraction, except for one patient (#17) who received hypofractionated SBRT for supraglottic laryngeal cancer on protocol. Mean age was 60.0 ± 8.9 yr.

**Table 1:** Patient demographics and treatment

Patient	Age	Primary site	Treatment coverage	Dose‐fractionation
1	53	Nasopharynx	Nasopharynx and bilateral neck with arc IMRT and matched AP low neck fields	6996 cGy in 33 fractions
2	82	Oropharynx	Oropharynx and bilateral neck with arc IMRT and matched AP low neck fields	6996 cGY in 33 fx
3	50	Oropharynx	Oropharynx and bilateral neck with arc IMRT and matched AP low neck fields	6600 cGy in 30 fx
4	63	Nasopharynx	Right nasopharynx with arc IMRT	6600 cGy in 30 fx
5	54	Oropharynx	Right base of tongue and bilatreal neck with arc IMRT with matched low AP neck fields.	6996 cGy in 33 fx
6	59	Larynx s/p Laryngectomy	Surgical bed in larynx and at risk lymph node regions in bilateral neck with IMRT.	6000 cGy in 30 fx.
7	41	Nasopharynx	Nasopharynx and bilateral neck treated with IMRT and matched low neck AP fields.	6996 cGy in 33 fx
8	60	Oropharynx	Right neck with IMRT.	6300 cGy in 30 fx
9	63	Thyroid	Surgical site and bilateral neck with IMRT.	6600 cGy in 30 fx.
10	51	Oropharynx	Oropharynx and bilateral neck with IMRT.	6300 cGy in 30 fx.
11	59	Cervical neck	Left face and neck with IMRT and low neck AP field.	6300 cGy in 30 fx
12	61	Oropharynx	Oropharynx with IMRT.	6996 cGy in 33 fx.
13	73	Hypopharynx	Hypopharynx, involved nodes, elective nodes with IMRT.	6996 cGy in 33 fx
14	60	Supraglottic larynx	Supraglottic larynx with IMRT.	6300 cGy in 30 fx.
15	57	Maxillary sinus	Paranasal sinus and neck with IMRT.	7000 cGy in 33 fx.
16	64	Oropharynx	Base of tongue with IMRT technique and neck tx with matched low neck fields.	6000 cGy in 30 fx.
17	60	Supraglottic larynx	Larynx and involved nodes with on‐protocol SBRT.	4250 cGy in 5 fx
18	65	Thyroid	Thyroid bed and cervical neck with IMRT.	6600 cGy in 30 fx.
19	54	Unknown primary	Neck treated with arc IMRT.	6300 cGy in 30 fx.
20	71	Hypopharynx	Hypopharynx and neck treated with arc IMRT.	6300 cGy in 30 fx

### 2.C Clinical workflow

**Figure 1:**
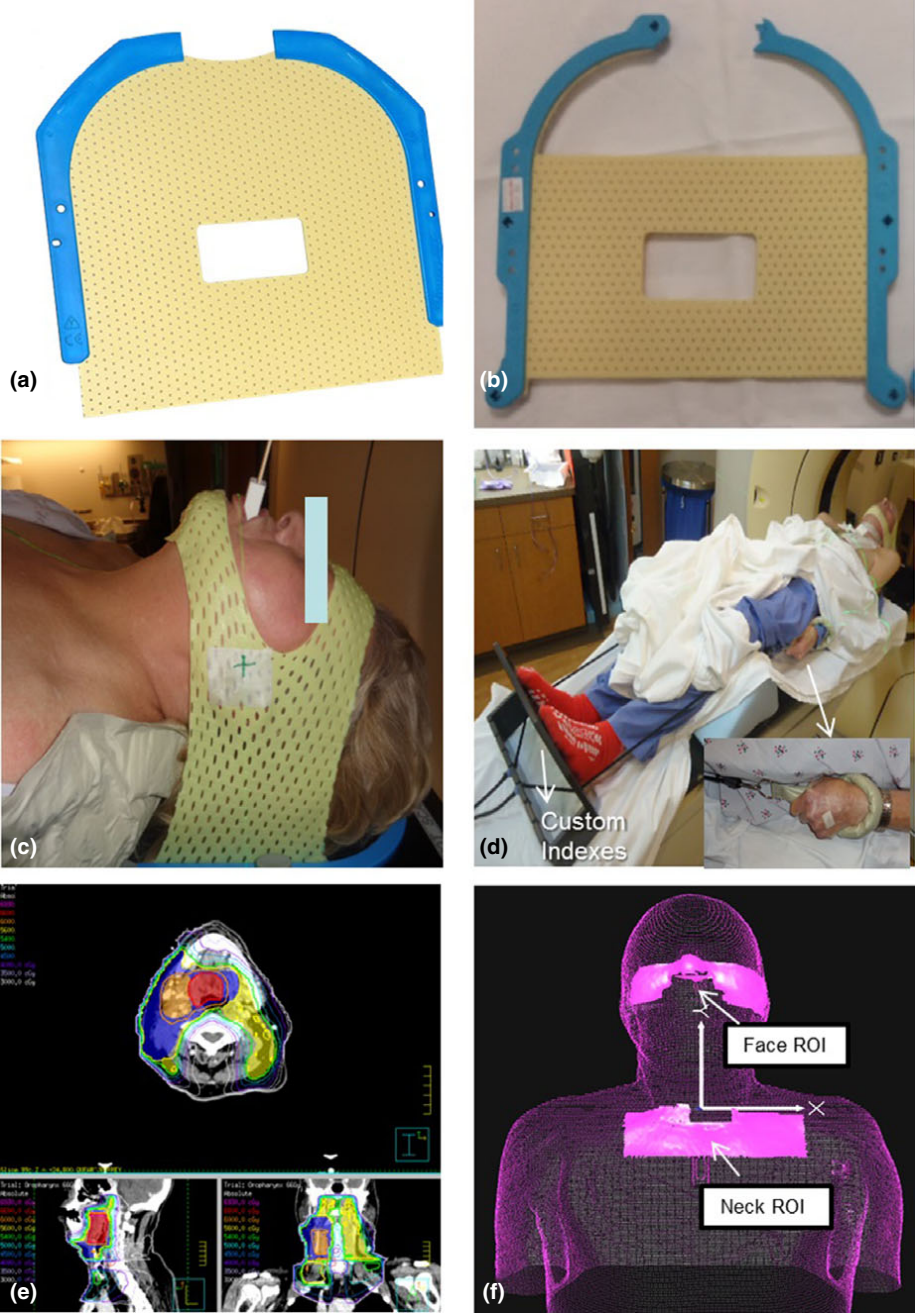
(a) Original short 3‐point mask (Qfix, model RT‐1876KSDGLF). (b) Mask modified with straight cuts at top and bottom. (c) Modified mask in place over only forehead and chin. (d) Overall patient setup. (e) Patient treatment plan with low neck coverage. (f) ROI selections on AlignRT relative to isocenter location. These two ROIs can be used to create a composite ROI for intrafractional tracking.

We modified commercial thermoplastic masks (Qfix, Avondale, Pennsylvania, USA, model RT‐1876KSDGLF) to immobilize only forehead and chin. The original mask has a precut 5 × 9 cm mid‐face opening [Fig. 1(a)]. The mask was further modified by removing horizontal strips from top and bottom leaving 5 cm of material above and below the opening [Fig. 1(b)]. Moldcare cushions (Qfix, 20 × 35 cm, model RT‐4492U) were fitted over a standard headrest (Qfix Q‐1). Shoulder movement was restricted by either: (a) a moldable cushion at the shoulders [Fig. 1(c)] in eight patients or (b) shoulder retractors (Civco, Orange City, Iowa, USA, model 20SR01SUB1) in 12 patients [Fig. 1(d)]. Mask coverage was insolated to chin and forehead [Figs. 1(c)and 1(d)]. Our standard IMRT planning employed two to four VMAT coplanar arcs with a matched AP low neck field [Fig. 1(e)].

Before the first day of treatment, skin surface data and CT images from simulation were imported into the Vision RT system. Patients were initially set up to surface markings verified by AlignRT. Two regions of interest (ROI) for SGRT monitoring were then selected: (a) nose and cheeks, (b) center neck strip excluding shoulders [Fig. 1(f)]. A mid‐neck ROI was not used due to interference from patient swallowing. A composite ROI can be generated from these two ROIs. Therapists first adjusted the head position based on the nose/cheek ROI in six dimensions (vertical, longitudinal, lateral, rotation, pitch, and roll). Error thresholds were set at a default value of ±1.5 mm for longitudinal, lateral, and vertical shifts and 1° for rotation, pitch, and roll. After this, the therapists then adjusted low neck/shoulder position using the neck ROI, with 3 mm shift and 3° rotation error thresholds to align the AP neck field. Therapists went back to nose/cheek ROI to ensure head position within default threshold. Therapists verified this full setup with routine in‐room CBCT imaging. Online CBCT matching first included bony spine and skull anatomy, followed by soft tissue matching around PTV. Setup error was evaluated in 6 degrees of freedom and documented. Repeat surface rendering information was acquired as a reference for subsequent treatments. Daily kV films were acquired for portal verification and physician review. Only one ROI and one threshold can be set at a time. Intrafraction motion was tracked on the combined ROIs (neck ROI + face ROI) with default threshold settings (1.5 mm/1°) during treatment with AlignRT. Standard clinic procedure called for treatment to hold for sustained movement beyond the error thresholds above. Repositioning and repeat on‐board orthogonal kV imaging was permitted, but not employed for any patient. For subsequent treatment days, therapists would use the previous day's surface rendering for initial setup. CBCT and OBI images were acquired as prescribed by the attending physician. At each treatment, therapists recorded total time from patient entry to exit from the treatment room. Beam gating was manually controlled by therapists.

### 2.D Patient comfort survey

Following simulation, patients completed a survey form to measure patient acceptance of the minimal mask. All four items were answered along the same 6‐point Likert scale: (a) unsure, (b) not at all, (c) a little bit, (d) somewhat, (e) quite a bit, (f) completely. The first item asked: “How comfortable was the mask?” The second item: “How securely did the mask keep you in one place?” The third item: “How confident are you that you will be able to tolerate this mask every day during treatment?” The fourth item: “How satisfied are you with this overall experience?” Patient survey data were tabulated and reported as raw values.

### 2.E Data analysis

Setup accuracy based on SGRT was compared against CBCT. Group mean and standard deviation were calculated for all treatment fractions from all patients. Systematic setup error(∑) and the random error (σ) were also calculated.^11^ Systematic error is the standard deviation of the individual patient means from his/her entire treatment fractions. Random error is the root mean square of the individual patient standard deviation from treatment fractions. These two measures are ingredients of popular margin recipe from Van Herk.^12^ Similarly these metrics were also performed on the intrafraction motion collected by AlignRT.

## 3 RESULTS

### 3.A Position verification

A total of 591 CBCTs were obtained for reference to SGRT. Average couch shifts following CBCT verification of SGRT‐guided setup are listed in Table 2. Also shown are systematic and random errors based on the two shoulder restriction methods we used, as well as for the cohort as a whole. Average shifts and errors were smaller with molded shoulder cushions vs shoulder retractors. Overall systematic error on translational shifts was small (<1.4 mm) and random error varied. Vertical displacements produced the largest random error, longitudinal errors were smallest. Approximately 5%–10% fractions were impacted by shifts >5 mm, 0%–3% fractions required >7 mm shifts. Rotational errors were small (<1°), with few setups requiring angle correction of 2° (7%–10%) or 3° (0.3%).

**Table 2:** Summary of setup shifts based on CBCT and intrafraction motion

Patient Cohort		Vrt (mm) (min, max)	Lng (mm) (min, max)	Lat (mm) (min, max)	Rotation (^o^) (min, max)	Pitch (^o^) (min, max)	Roll (^o^) (min, max)
Setup shift based on CBCT (mm or ^o^)							
Shoulder cushion	Group average	−0.73 ± 2.08 (−8.0, 7.0)	−0.21 ± 2.75 (−7.0, 8.0)	0.17 ± 2.68 (−8.0, 10.0)	−0.13 ± 0.87 (−3.6, 3.0)	−0.24 ± 0.97 (−3.0, 2.9)	0.13 ± 0.87 (−3.0, 3.0)
	∑	0.71	0.66	0.68	0.29	0.25	0.27
	σ	2.70	2.65	2.54	0.84	0.95	0.84
Shoulder retractors	Group average	−0.35 ± 2.60 (−11.1, 11.0)	−0.70 ± 3.65 (−9.0, 10.0)	0.28 ± 2.96 (−14.0, 11.0)	−0.16 ± 1.10 (−3.0, 3.0)	0.14 ± 1.31 (−2.8, 3.0)	0.02 ± 1.21 (−3.0, 3.0)
	∑	1.37	0.95	1.12	0.54	0.69	0.47
	σ	3.46	2.80	2.94	1.00	1.11	1.14
Total cohort	Group average	−0.51 ± 2.42 (−11.1, 11.0)	−0.49 ± 3.30 (−9.0, 10.0)	0.23 ± 2.58 (−14.0, 11.0)	−0.15 ± 1.01 (−3.6, 3.0)	−0.02 ± 1.19 (−3.0, 3.0)	0.06 ± 1.08 (−3.0, 3.0)
	∑	1.18	0.90	0.98	0.46	0.58	0.41
	σ	3.18	2.74	2.79	0.94	1.05	1.03
Intrafraction motion (mm or ^o^)							
Shoulder cushion	Group average	−0.02 ± 0.74	−0.04 ± 0.87	0.04 ± 1.01	−0.05 ± 0.48	−0.03 ± 0.40	−0.05 ± 0.41
	∑	0.15	0.15	0.13	0.08	0.09	0.11
	σ	0.69	0.81	0.82	0.44	0.35	0.40
Shoulder retractors	Group average	0.24 ± 1.00	−0.16 ± 0.78	0.10 ± 0.80	0.00 ± 0.40	−0.02 ± 0.42	−0.11 ± 0.44
	∑	0.36	0.26	0.21	0.11	0.12	0.16
	σ	0.79	0.70	0.75	0.37	0.39	0.40
Total cohort	Group average	0.11 ± 0.89	−0.10 ± 0.83	0.07 ± 0.92	−0.02 ± 0.44	−0.02 ± 0.41	−0.08 ± 0.43
	∑	0.30	0.24	0.18	0.10	0.11	0.14
	σ	0.75	0.75	0.78	0.40	0.38	0.40

**Figure 2:**
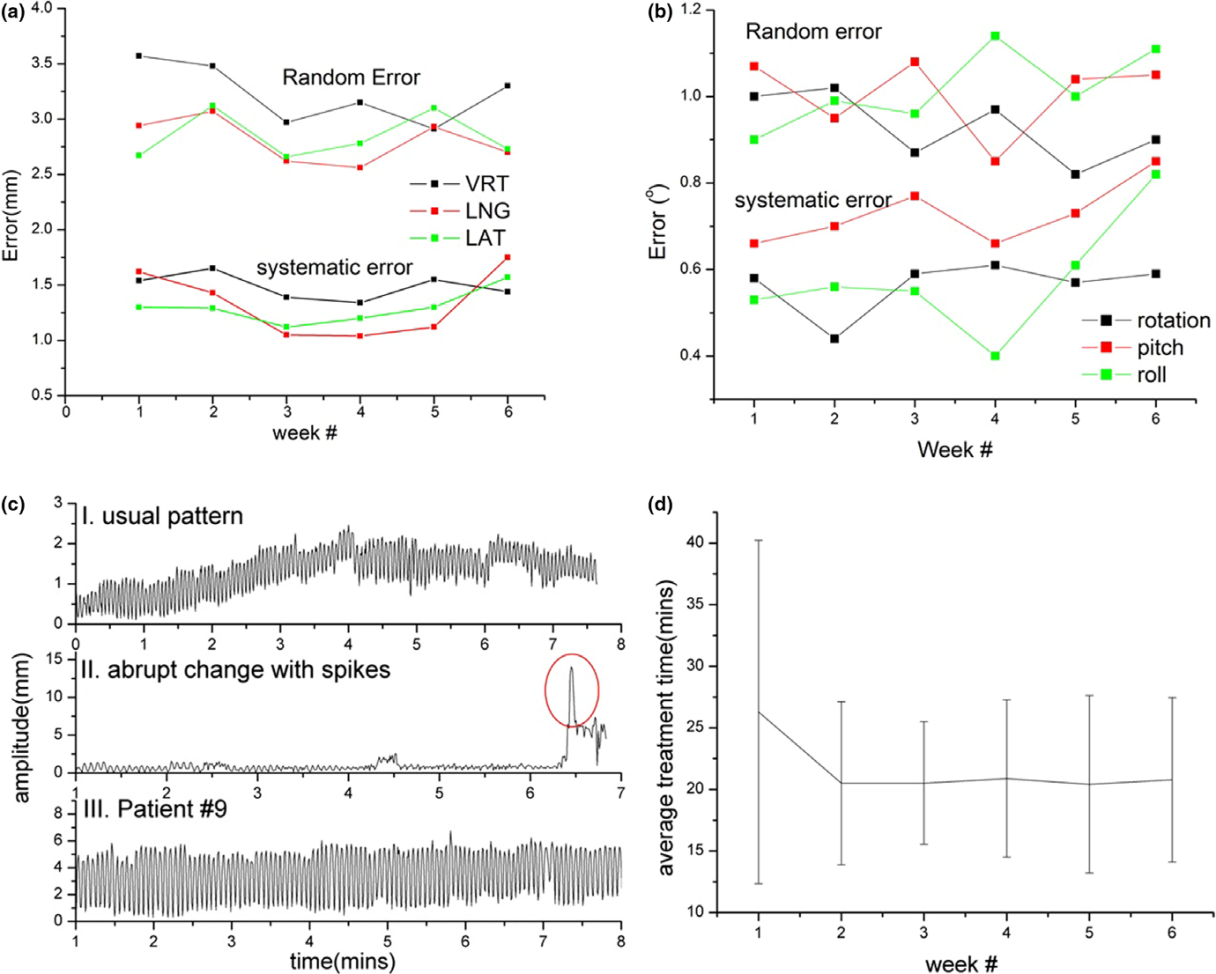
System and random errors plotted by week for (a) translational and (b) rotational shifts; (c) examples of intrafraction motion patterns (see text for details); (d) total in‐room treatment time per fraction plotted across each week.

Setup errors across treatment weeks are plotted longitudinally in Fig. 2. Translational and rotational errors settled after the first 2 weeks. Interestingly, errors spiked backup to first week levels during the final week of treatment, potentially reflecting cumulative impact of anatomic changes across the treatment course. Incidentally, nearly all patients lost weight by end of treatment. Total weight loss averaged 5.3 ± 4.7 kg. Nonetheless, magnitude of systematic and random errors remained small across all time points.

### 3.B Intrafraction motion

Similar analysis was performed for intrafraction motion data. A total of 596 treatments were analyzed. As shown in Table 2, average motion and errors were small (<1 mm). Differences between the two shoulder restriction methods were not significant (*P *= 0.31). Magnitude of error was not time‐dependent across treatment weeks. Three examples of the intrafraction motion pattern were shown in Fig. 2(c), plotted as amplitude change with time. Detailed case‐by‐case analysis confirmed small motion amplitude (<1 mm) across the entire cohort; nonetheless, range of error was larger in a minority of cases. Top and bottom plots are examples of patients who kept their setup position and breathed smoothly. Slight baseline drift can be observed; treatment was continued in both cases since this was felt to represent natural respiratory motion of the patient. For example, raw surface imaging data for Patient #9 confirmed extended respiration motion at upper neck ROI. This motion amplitude remained less than ~4 mm and did not impact clinical setup quality of our conventional AP low neck fields. The middle plot demonstrates a patient who displayed acute displacement due to coughing (red circle). Therapists immediately stopped treatment and waited for the patient to settle and for respiratory motion to return to baseline; patient position was then manually checked by the therapist in‐room before treatment was restarted. The majority of patients completed all treatments without interruption.

### 3.C In‐room setup and treatment time

Average total treatment time with complete SGRT guidance was 21.6 ± 8.4 min, closely matching a standard 20‐min treatment time slot for head and neck IMRT. Required time was not statistically different between the two methods (*P *= 0.14). Treatment time settled after the first week [Fig. 2(d)], averaging 20.6 min at week 2 and beyond.

### 3.D Patient comfort survey

A total of 19 of 20 surveys were returned. We specifically desired patients to provide stand‐alone assessments of the minimal masks; no study patient tried a regular fully closed mask for comparison. Out of a maximum score of 6, average score for mask comfort was 5.11 ± 0.81, average patient perception of secure immobilization was 5.21 ± 0.71, average patient confidence of continued mask tolerability was 5.63 ± 0.60, and average overall patient satisfaction score was 5.37 ± 1.30. The answers for each question were close (within 2) for each individual patient except for patient #19 who put value “unsure” for last question and hence had a large standard deviation (1.30).

## 4 DISCUSSION

In this study, we present a streamlined workflow using minimal mask face coverage in combination with optical surface guidance for treatment of head and neck cancer patients with setup errors, intrafractional motion, and treatment times comparable to standard mask immobilization. Previous studies have described several open mask solutions. These trials mostly focus on brain SRS treatment^5^ or lesions in head that requires head mask only.^9^ Velec et al. described a long mask modified with opening at neck to reduce skin dose.^13^ Patients were positioned using skin marks with no intrafraction tracking. Wiant et al. described another long mask solution with a small facial opening for H&N treatment.^8^ This mask is commercially available (Openview Assure, Qfix, Avondale, PA, USA). Exact use of surface imaging was not fully described. Our open mask solution yields minimal coverage to the skin compared with commercially available mask, is easily fabricated by hand without special tools, and provides a high level of patient comfort.

**Figure 3:**
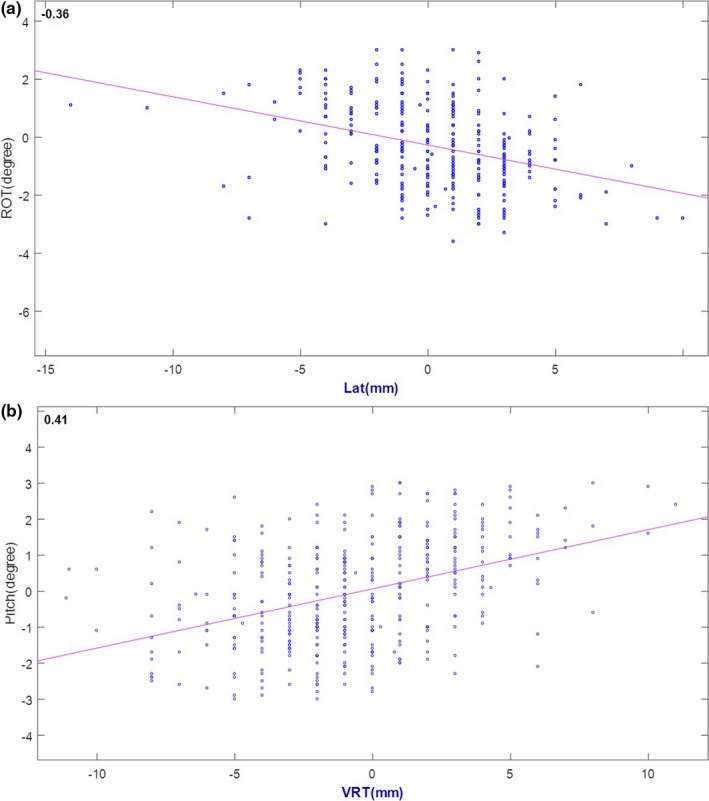
Correlation between angular correction and translational shift: (a) rotation and lateral shift, (b) pitch and vertical shift.

Most studies report translational set up errors ranging between 0 and 3.6 mm for systematic errors and 1.0–2.6 mm for random errors.^8^, ^13^, ^14^, ^15^, ^16^, ^17^, ^18^ Few studies report angle corrections, undoubtedly because six‐dimensional robotic couches are a relatively new commercial offering. Although systematic errors were small, we observed random errors at the larger end of reported values, potentially due to correction with our six‐dimensional couch. Angle correction can be complicated by interplay between rotational and translational corrections due to distance between isocenter and the center of an image matching box, as noted by Den et al.^14^ Figure 3 shows the correlation plot between angle correction and translation shift: (a) rotation and lateral shift, (b) pitch and vertical shift. Our limited data have shown weak correlation (*r* = 0.36–0.41) between angle correction and translation shift. Furthermore, the location of the matching box varies with location of lesions being treated, causing differences in registration.^17^, ^18^ Some studies mention manual repositioning of patients if initial x‐ray imaging indicate need for a large angle correction of ≥3–5°, but this is of course without confirmation of such errors with surface‐based imaging.^14^, ^16^ In our study, therapists corrected angle errors only if corroborated by real‐time surface guidance; thus, our reported data provide direct comparison between surface and x‐ray image guidance. Rotational setup errors were limited to ~0.5^o^∑ and ~1.0^o^ *σ* (Table 2) with surface guidance. Therefore, the need for angle corrections with an expensive six‐dimensional robotic couch is limited. Treatment time with our minimal mask platform is already comparable to standard closed mask treatment time.

Intrafractional motion in our study was also small. We used a composite ROI which included face and neck to cover the entire treatment area. Six‐dimensional intrafractional motion tracking with this ROI was comparable to previous studies.^15^ Reports have shown that tracking a face ROI alone detects only small positional deflections (<1 mm). Respiratory neck motion is a more likely source of larger motion during treatment; this is supported by our detection of sinusoidal motion patterns [Fig. 2(c)].

Another component of our study included assessment of shoulder immobilization methods. We compared two shoulder restriction methods and found that a moldable cushion provides better setup than shoulder stirrups. With the moldable cushion, patients are able to rest their arms and elbows at anchoring points, which makes shoulder and neck position more reproducible.

Interestingly, we found that translational and rotational errors spiked during the final week of treatment (Fig. 2). Total patient weight loss averaged >5 kg during treatment. Significant weight loss accumulating during the final weeks of treatment may impact daily setup position and reproducibility.^14^

Some of the limitations of our study included an inability to separately track distinct face and neck ROIs simultaneously. The AlignRT software only allowed one ROI to be tracked at a time. Therefore, therapists had to set up the face and neck separately. These ROIs tend to move together, creating novel setup challenges for some patients. Since head setup through surface guidance is more reproducible (as shown in prior studies on brain SRS setups), we observed therapists focusing on face/head positioning throughout their setup process.

Therapists had the option to use DICOM or last captured surface as reference. The benefit of DICOM surface is to have consistent “DRR” during surface setup. In this pilot study, we decided to use last captured surface as primary reference surface of the next day as an analogy of established surface‐guided breast setup. The face ROI is relatively reproducible because of rigid head. We assumed updating reference surface would help incorporate patient positional change below head caused by immobilization device deformation and weight change over time. Future work will track HN deformation based on captured surfaces over a course of treatment. We did not single out the setup accuracy due to minimal mask immobilization and surface guidance. Currently, we continue to consider daily CBCT as our reference standard for image guidance.

It is important to emphasize that primary objective of this pilot study was to confirm feasibility of minimal coverage immobilization for patients normally confined by large uncomfortable masks. SGRT was a secondary image guidance maneuver employed to: (a) ensure fidelity of initial patient set up and (b) track motion during treatment. Although set up accuracy was comparable to that of standard coverage masks for patients with similar treatment anatomy,^8^, ^13^, ^14^, ^15^, ^16^, ^17^, ^18^ surface guidance alone is not enough to ensure reproducible daily setup given the large translational random setup errors. Therefore, on‐board imaging is recommended to confirm setup. Considering small rotational setup errors, planar KVs may be sufficient.

Finally, although patient‐reported comfort appeared to be high, we did not conduct a direct comparison between minimal vs standard mask comfort in the study patients. This was intentional, since we wished to minimize patient bias toward higher comfort scores for the minimal mask after trying a standard mask on. Future studies may directly compare patient comfort between both systems.

## 5 CONCLUSION

We present a minimal mask immobilization solution with a streamlined clinical workflow for treatment with surface guidance. Surface guidance facilitates patient motion tracking during treatment and provides modest help in pretreatment set up. On‐board radiographic imaging remains our recommended standard. Compared with other open mask methods, our solution offers patients minimal face coverage and optimal comfort. We have also provided our step‐by‐step workflow as a practical guideline for implementation.

## CONFLICT OF INTEREST

This study was supported by Vision RT, Ltd. (London, UK). 

## References

[1] Lee N , Chuang C , Quivey JM , et al. Skin toxicity due to intensity‐modulated radiotherapy for head‐and‐neck carcinoma. Int J Radiat Oncol Biol Phys. 2002;53:630–637. 12062606 10.1016/s0360-3016(02)02756-6

[2] Peng JL , Kahler D , Li JG , et al. Characterization of a real‐time surface image‐guided stereotactic positioning system. Med Phys. 2010;37:5421–5433. 21089778 10.1118/1.3483783

[3] Kelly A , Hardcastle N , Metcalfe P , et al. Surface dosimetry for breast radiotherapy in the presence of immobilization cast material. Phys Med Biol. 2011;56:1001. 21258139 10.1088/0031-9155/56/4/008

[4] Krengli M , Gaiano S , Mones E , et al. Reproducibility of patient setup by surface image registration system in conformal radiotherapy of prostate cancer. Radiat Oncol. 2009;4:1. 19232137 10.1186/1748-717X-4-9 PMC2649941

[5] Cerviño LI , Detorie N , Taylor M , et al. Initial clinical experience with a frameless and maskless stereotactic radiosurgery treatment. Pract Radiat Oncol. 2012;2:54–62. 24674037 10.1016/j.prro.2011.04.005

[6] Pan H , Cerviño LI , Pawlicki T , et al. Frameless, real‐time, surface imaging‐guided radiosurgery: clinical outcomes for brain metastases. Neurosurgery. 2012;71:844–852. 22989959 10.1227/NEU.0b013e3182647ad5

[7] Alderliesten T , Sonke J‐J , Betgen A , van Vliet‐Vroegindeweij C , Remeijer P . 3D surface imaging for monitoring intrafraction motion in frameless stereotactic body radiotherapy of lung cancer. Radiother Oncol. 2012;105:155–160. 23026398 10.1016/j.radonc.2012.08.016

[8] Wiant D , Squire S , Liu H , Maurer J , Hayes TL , Sintay B . A prospective evaluation of open face masks for head and neck radiation therapy. Pract Radiat Oncol. 2016;6:e259–e267. 27025164 10.1016/j.prro.2016.02.003

[9] Li G , Lovelock DM , Mechalakos J , et al. Migration from full‐head mask to open‐face mask for immobilization of patients with head and neck cancer. J Appl Clin Med Phys. 2012;14:243–254. 10.1120/jacmp.v14i5.4400 PMC5714571 24036878

[10] Schreibmann E , Elder E , Fox T . Automated quality assurance for image‐guided radiation therapy. J Appl Clin Med Phys. 2009;10:2919. 19223842 10.1120/jacmp.v10i1.2919 PMC5720496

[11] van Herk M . Errors and margins in radiotherapy. Paper presented at: Seminars in radiation oncology. 2004. 10.1053/j.semradonc.2003.10.003 14752733

[12] van Herk M , Remeijer P , Rasch C , Lebesque JV . The probability of correct target dosage: dose‐population histograms for deriving treatment margins in radiotherapy. Int J Radiat Oncol Biol Phys. 2000;47:1121–1135. 10863086 10.1016/s0360-3016(00)00518-6

[13] Velec M , Waldron JN , O'Sullivan B , et al. Cone‐beam CT assessment of interfraction and intrafraction setup error of two head‐and‐neck cancer thermoplastic masks. Int J Radiat Oncol Biol Phys. 2010;76:949–955. 20056344 10.1016/j.ijrobp.2009.07.004

[14] Den RB , Doemer A , Kubicek G , et al. Daily image guidance with cone‐beam computed tomography forâ head‐and‐neck cancer intensity‐modulated radiotherapy: aâ prospective study. Int J Radiat Oncol Biol Phys. 2010;76:1353–1359. 19540071 10.1016/j.ijrobp.2009.03.059

[15] Pang PPE , Hendry J , Cheah SL , et al. An assessment of the magnitude of intra‐fraction movement of head‐and‐neck IMRT cases and its implication on the action‐level of the imaging protocol. Radiother Oncol. 2014;112:437–441. 25284062 10.1016/j.radonc.2014.09.008

[16] Vaandering A , Lee JA , Renard L , GrÃ©goire V . Evaluation of MVCT protocols for brain and head and neck tumor patients treated with helical tomotherapy. Radiother Oncol. 2009;93:50–56. 19515441 10.1016/j.radonc.2009.05.009

[17] van Kranen S , van Beek S , Rasch C , van Herk M , Sonke J‐J . Setup uncertainties of anatomical sub‐regions in head‐and‐neck cancer patients after offline CBCT guidance. Int J Radiat Oncol Biol Phys. 2009;73:1566–1573. 19306753 10.1016/j.ijrobp.2008.11.035

[18] Zhang L , Garden AS , Lo J , et al. Multiple regions‐of‐interest analysis of setup uncertainties for head‐and‐neck cancer radiotherapy. Int J Radiat Oncol Biol Phys. 2006;64:1559–1569. 16580505 10.1016/j.ijrobp.2005.12.023

